# Carvacrol: From Ancient Flavoring to Neuromodulatory Agent

**DOI:** 10.3390/molecules18066161

**Published:** 2013-05-24

**Authors:** Margherita Zotti, Marilena Colaianna, Maria Grazia Morgese, Paolo Tucci, Stefania Schiavone, Pinarosa Avato, Luigia Trabace

**Affiliations:** 1 Department of Clinical and Experimental Medicine, University of Foggia, Foggia 71121, Italy; E-Mails: m.zotti@unifg.it (M.Z.); m.colaianna@unifg.it (M.C.); mg.morgese@gmail.com (M.G.M.); p.tucci@unifg.it (P.T.); 2 Department of Pathology and Immunology, University of Geneva, Geneva 1211, Switzerland; 3 Department of Mental Health and Psychiatry, Geneva University Hospital and University of Geneva, Geneva 1211, Switzerland; E-Mail: stefania.schiavone@unige.ch; 4 Department of Pharmacy-Drug Sciences, University of Bari, “A. Moro”, Bari 70125, Italy; E-Mail: pinarosa.avato@uniba.it

**Keywords:** carvacrol, dopamine, serotonin, behavior

## Abstract

Oregano and thyme essential oils are used for therapeutic, aromatic and gastronomic purposes due to their richness in active substances, like carvacrol; however, the effects of the latter on the central nervous system have been poorly investigated. The aim of our study was to define the effects of carvacrol on brain neurochemistry and behavioural outcome in rats. Biogenic amine content in the prefrontal cortex and hippocampus after chronic or acute oral carvacrol administration was measured. Animals were assessed by a forced swimming test. Carvacrol, administered for seven consecutive days (12.5 mg/kg p.o.), was able to increase dopamine and serotonin levels in the prefrontal cortex and hippocampus. When single doses were used (150 and 450 mg/kg p.o.), dopamine content was increased in the prefrontal cortex at both dose levels. On the contrary, a significant dopamine reduction in hippocampus of animals treated with 450 mg/kg of carvacrol was found. Acute carvacrol administration only significantly reduced serotonin content in either the prefrontal cortex or in the hippocampus at the highest dose. Moreover, acute carvacrol was ineffective in producing changes in the forced swimming test. Our data suggest that carvacrol is a brain-active molecule that clearly influences neuronal activity through modulation of neurotransmitters. If regularly ingested in low concentrations, it might determine feelings of well-being and could possibly have positive reinforcer effects.

## 1. Introduction

Carvacrol [2-Methyl-5-(1-methylethyl) phenol] is the major natural constituent in the essential oil fraction of aromatic plants belonging to the family Lamiaceae, such as oregano and thyme. It is a monoterpenic phenol biosynthesized from γ-terpinene, through *p*-cymene and containing methyl and isopropyl functions in the *para* position to each other on a phenol ring [1].

Carvacrol has been approved by the Food and Drug Administration for food use and it is included by the Council of Europe in the list of approved chemical flavorings [2,3]. The biological activities of oregano, actually used in food, spice and pharmaceutical industries, were suggested to parallel the carvacrol content [4]. Drugs and/or essential oils containing carvacrol have been widely used in traditional medicine, and a large number of feed additives based on this molecule are at present commercially available [5]. Moreover, an experimental study of mental attitudes among general practitioners and the general population with respect to the use of pharmaceuticals and natural remedies by healthy people demonstrated that natural remedies were more acceptable to the general public than the use of pharmaceuticals for improving concentration, mood and memory [6]. However, in spite of the widespread use of plant-derived metabolites, such as carvacrol, to improve mood and cognition in healthy individuals, additional data to clarify the mechanism of action are needed.

Several reports indicated that carvacrol exhibits fungicidal [7,8], insecticidal [9] and antimicrobial activities [10]. *In vitro* studies attributed to carvacrol anticarcinogenic and antitumor activities [11,12], “strong antimutagenic effects” and antioxidant properties [13,14]. A recent study showed that this phytochemical protects liver against ischemia/reperfusion (I/R) injury in rats [15]. Due to its lipophilicity and capacity to readily cross membranes, such as the blood-brain barrier [16], this volatile molecule can accumulate in the brain, interacting with different receptor sites in the central nervous system (CNS) showing centrally active properties [17,18]. However, up to now, few and sporadic studies have been carried out on its *in vivo* effects on the CNS. Recent findings showed that carvacrol inhibits the Transient Receptor Potential (TRP) Cation channel, subfamily M, member 7 (TRPM7) of the TRP family expressed in both HEK cells and CA3–CA1 hippocampal primary cultured cells. It was demonstrated that TRPM7 is an essential mediator of anoxic neuronal death [19]. Therefore, inhibition of TRPM7 is expected to reduce cell death following ischemia and brain damage. It has been recently shown that carvacrol may play a role to protect against cerebral I/R injury [20]. Moreover, carvacrol exerts a strong acetylcholinesterase (AChE) inhibitory effect due to the molecule hydroxyl group binding to AChE, causing a loss of function of AChE, thus suggesting a possible application in Alzheimer’s disease treatment [21]. In addition, recent experimental data indicated that carvacrol induces anxiolytic-like effect and antidepressant-like properties in tasks for anxiety and depression behaviour in mice [22,23]. It is likely that the bioactivity of this specific compound, having the ability to modulate mood and cognitive processes, involves several neurotransmitter systems in the brain. To this end, in the present study *in vivo* and *ex-vivo* investigations were performed in rats to clarify the involvement of neurotransmitter systems that control affective state and emotional responses, in the pharmacological action of carvacrol. In particular, possible alterations induced by low chronic and high acute doses of carvacrol on monoaminergic transmission were investigated in the prefrontal cortex (PFC) and hippocampus (HIPP) of male Wistar rats. These two ranges of treatments were chosen in order to partially mimic carvacrol accumulation after daily consumption, in one case, and to investigate on a possible acute effect in the other one. Moreover, the effects of this compound were analysed in a behavioural test sensitive to antidepressant-like activity.

## 2. Results and Discussion

**Figure 1:**
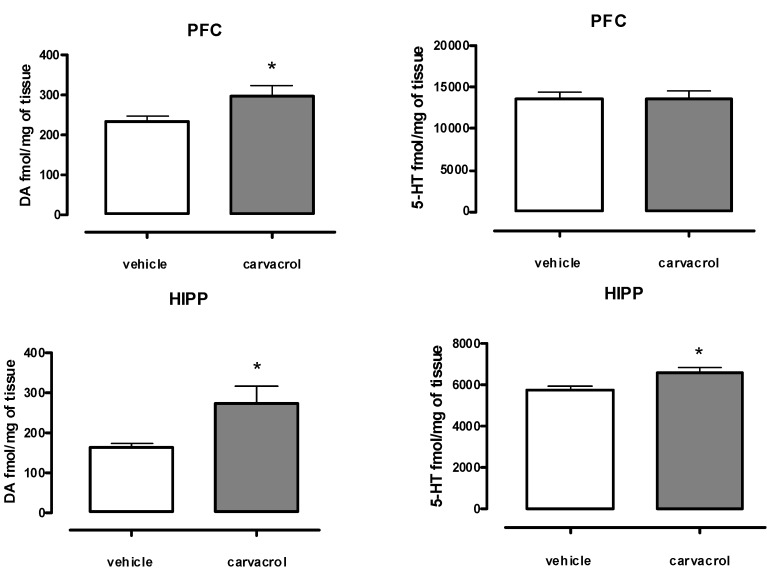
Measurement of DA and 5-HT content in PFC and HIPP of male Wistar rats, orally treated with carvacrol (7 consecutive days, 12.5 mg/kg) or vehicle (peanut oil, 1 mL/kg). Data were expressed as mean + S.E.M. (*n =* 8, unpaired student’s t-test * *P* < 0.05).

Oral administration of 12.5 mg/kg of carvacrol for seven consecutive days caused a significant increase in dopamine (DA) tissue content, either in PFC or in the HIPP, with respect to controls (*P* < 0.05; Figure 1). Moreover, subchronic treatment with carvacrol induced a significant increase in serotonin (5-HT) levels only in the HIPP (*P* < 0.05; Figure 1).

**Figure 2:**
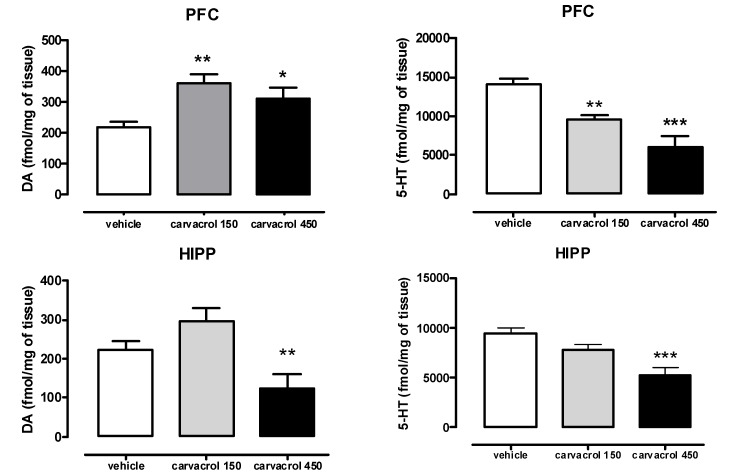
Measurement of DA and 5-HT content in PFC and HIPP of male Wistar rats orally treated with carvacrol (acute administration, 150 or 450 mg/kg) or vehicle (peanut oil, 1 mL/kg). Data were expressed as mean + S.E.M. (*n =* 8, one-way Anova, followed by Tukey’s test, * *P* < 0.05, ** *P* < 0.01, *** *P* < 0.001).

As shown in Figure 2, acute administration of carvacrol (150 and 450 mg/kg p.o.) significantly increased DA content in the PFC at both tested doses (F(2,22) = 8.514; ** *P* < 0.01 and * *P* < 0.05). On the contrary, a significant reduction of DA tissue content in HIPP of animals treated with 450 mg/kg of carvacrol was found (F(2,26) = 6.822, ** *P* < 0.01). As far as serotonergic system, carvacrol administration significantly reduced 5-HT content either in PFC (F(2,18) = 19.85; ** *P* < 0.01 and *** *P* < 0.001) or in HIPP at 450 mg/kg (F(2,21) = 11.70; *** *P* < 0.001).

**Figure 3:**
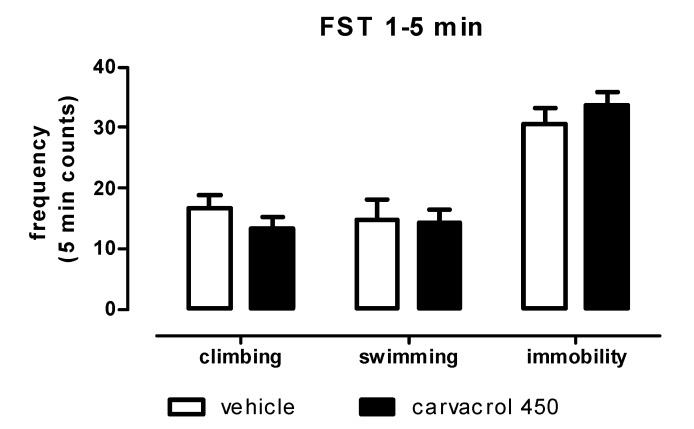
Measures of climbing, swimming and immobility frequencies during FST in male Wistar rats after carvacrol (450 mg/kg, p.o.) or vehicle (peanut oil, 1 mL/kg, p.o.) administration. Data were expressed as mean + SEM (*n* = 10, unpaired student’s t-test, n.s.).

In order to study if neurochemical alterations were accompanied by behavioural outcomes, a forced swimming test (FST) was performed. In this behavioural paradigm, carvacrol was tested only at the higher dose (450 mg/kg p.o.), which was found able to evoke alterations on both neurotransmitters in the two brain areas considered in the study. Carvacrol was ineffective in producing changes in immobility, swimming or climbing activities during the test compared to controls animals (Figure 3). 

Our results from this study provide evidence for a modulatory activity of carvacrol on dopaminergic and serotonergic transmissions in two cerebral areas of male Wistar rats. Due to its low molecular weight (150.2 g/mol) and its lipophilic profile, carvacrol is believed to easily and quickly cross the blood–brain barrier [18,24]. The neuromodulatory activity shown in the present study can be taken as an indirect evidence to prove it. Accordingly, a direct evidence that carvacrol is a brain-active natural compound was reported in a recent study, where carvacrol showed neuroprotective properties when injected intracerebroventricularly, in a middle cerebral artery occlusion mouse model [20].

In the last years, human exposure to aromatic plant-based products in the Western world has expanded as a result of their increased application in dietary supplements and alternative medicine [25]. Carvacrol has been used on a large scale in the food and cosmetic industries, thus being a common ingredient of the human diet. Due to its potent organoleptic properties, this flavoring substance is typically added in low amounts to human food. For example, in non-alcoholic beverages, carvacrol is normally used up to a level of 26 ppm and in baked goods up to 120 ppm [26] and has received the generally recognized as safe substance status from the Flavor and Extract Manufacturers’ Association and the Food and Drug Administration of the USA [26]. Even if for carvacrol there exists a wide margin of safety, investigations on its properties are becoming important because of the increased human exposure to the compound. As far as oregano is concerned, more than 60 species are used as spices with great differences in their contents of aromatic volatile compounds [27]. Despite the large use of oregano oil in food, beverages and cosmetics, few data are available in the literature concerning acute and chronic effects, especially in vivo, of this substance [28,29,30]. Animals were orally tested with an acute and a subchronic dose regimen of carvacrol. In particular, a single gavage administration at two different doses, 150 and 450 mg/kg, corresponding to about 20% and 50% of the observed LD_50_ value *per os* in rats [31]. Results indicated that rats treated with carvacrol displayed impaired serotonergic transmission in both the brain areas considered in our study. Moreover, carvacrol significantly reduced DA levels in HIPP, while increased levels of DA were observed in PFC.

It is well known that DA and 5-HT play an essential role in many of the basic physiological and behavioral activities [32,33]. In particular, 5-HT is involved in the regulation of peripheral and central functions. Among them, 5-HT plays a crucial role in controlling mood [34], aggression [35], sexual behavior, pain sensitivity and learning [32], cardiovascular regulation, thermoregulation, as well as circadian rhythm, sleep–wake cycle and food intake [36]. On the other hand, DA is implicated in the regulation of cognitive processes, emotion [37], learning and memory, locomotor activity [38], endocrine regulation [37] and positive reinforcement [39]. Therefore, dysregulation of these aminergic neurotransmitter pathways is strongly implicated in alteration of mood, appetite, sleep, sexual, cognitive and emotional processes.

It was recently demonstrated by our group that, in female rats, the acute administration of carvacrol, at the dose of 450 mg/kg p.o., significantly reduced 5-HT content in the PFC and *nucleus accumbens*. Moreover, from a behavioral point of view, when animals were placed in an inescapable situation, such as that which occurs in the FST, carvacrol administration was effective in producing an increase in immobility duration, a condition which reflects depressant-like activity. This effect was reverted by pretreatment with the antidepressant fluoxetine, thus confirming the depressive-like properties of carvacrol in these conditions [18]. In the present study, although a neuromodulatory effect was observed, oral administration of the same dose of carvacrol surprisingly failed to induce changes in behavioral outcomes when male rats were assessed in the FST. The apparent lack of association between 5-HT reduction and the development of depression-like behavior in the FST, in male rats, may indicate the intervention of secondary processes or the involvement of other systems in the mechanism induced by carvacrol. In this regard, it is worthy to note that the monoamine hypothesis of depression is based on the deficiency of more than one monoamine [40] and most of the antidepressants exert their primary biochemical effects by regulating synaptic concentrations of several neurotransmitters, among which a pivotal role is certainly played by 5-HT and DA [41].

In particular, to interpret our data, one explanation might lie within the specific increase of DA levels in PFC. Such increase could derive from the disinhibition of the tonic inhibitory control of 5-HT on DA release in the respective projection regions such as PFC. Indeed, it has been reported that new antidepressant drugs exert their action by reducing 5-HT and by raising DA in the PFC [42].

To clarify the effects of carvacrol on brain monoamines, several hypotheses can be provided. Dysregulation of DA and 5-HT transmission could derive from alteration of transporter proteins or by influencing those mechanisms which are involved in the reuptake or metabolism of neurotransmitters. In particular, the oregano extract was demonstrated to inhibit the reuptake and degradation of the monoamine neurotransmitters in a dose-dependent manner, In addition, oregano extract dose dependently and reversibly inhibited the enzymatic activity of MAO-A [24]. The complementary effects of inhibiting both reuptake of the monoamine neurotransmitters and their enzymatic degradation would result in greater synaptic monoamine levels. Beyond this hypothesis, the mechanism through which carvacrol affects DA and 5HT system, by influencing their biosynthesis, release, or metabolism, remains still under investigation and further investigation are certainly required.

Accordingly, a recent study by Mechan and co-workers showed that oregano extract (principal volatile component identified as carvacrol: 50–79.9%), when orally administered to male mice in a dosing regimen 24, 5 and 1 h before the start of the FST, reduced immobility behavior and the significant effect was not observed above a dose of 150 mg/kg [24]. Thus, it could be postulated that, under our conditions, in male animals, such behavioral response appears within a specific dose range and this might be a saturable effect in *in vivo* conditions.

As known, botanicals are more acceptable by humans than pharmaceutical molecules. Then, in order to partially mimic carvacrol accumulation after daily consumption as a dietary additive, and to ensure an adequate intake, male rats were treated for seven consecutive days with an oral dose of 12.5 mg/kg. In our subchronic conditions, carvacrol administration induced a significant increase of DA levels either in HIPP or in PFC, while 5-HT content was enhanced in HIPP. Moreover subchronic administration of carvacrol was able to increase DA content in the nucleus accumbens (unpublished observations). Taking into account the role of DA substrates for incentive motivation, this stimulatory activity of DA systems in key brain areas leads to the hypothesis that this molecule could possibly have positive reinforcer effects, at least from a behavior-based perspective. Thus, our subchronic data seem to suggest that carvacrol, present in the essential oils of oregano plant, if regularly ingested in low concentrations through the diet might determine feeling of well-being, such as reward. 

Many research reports support the role of other terpenoid molecules in improving mental well-being in humans because they are brain-accessible, brain-active and clearly influence neuronal activity through modulation of neurotransmitter [43,44] and proposed the supplementation of traditional medicine with these active phytochemicals.

Other natural compounds and pharmaceuticals share with carvacrol similar effects on dopaminergic and serotonergic systems. For example, it has been shown that hypericin, a predominant component of *Hypericum perforatum*, given in 0.4–2.7 mg/day for 4–6 weeks to patients with mild to moderate depression, produces similar treatment response compared to traditional antidepressants drugs [45]. It has been suggested that the mechanism of action of hypericin relies on the inhibition of reuptake of 5-HT and DA, thus elevating their synaptic concentrations and this effect contributes to the plant's antidepressant action. Moreover, chronic but not acute administration of *Ginko biloba* extract results in increased dopaminergic transmission in the frontocortical brain areas, which may be one of the underlying mechanisms behind the clinically observed effects of *Ginko biloba* on improved cognitive function [46]. 

*Ginko biloba* extracts, besides their reported neuroprotective effects and improved memory function, may possess mood/motivation enhancing activities in disorders associated with abnormal monoaminergic and, in particular, dopaminergic function. Substances of abuse influence the limbic reward system by increasing the brain's levels of free DA in a dose-dependent manner; that is, more DA is available when higher doses of the substance are administered [47]. Indeed, high levels of free DA in the brain generally enhance mood and help to regulate the feelings of pleasure. Amphetamine and methamphetamine, in particular, are powerful acute stimulants. They act as indirect agonists, causing the release of newly synthesized monoamines in the brain and blocking the reuptake of these transmitters from the synapse [48]. The amphetamines are potent inhibitors of monoamine oxidase. Collectively, these effects lead to an increase in the concentration of aminergic in the synapse as well as an overall increase in aminergic activity in the brain [48]. In addition to their actions on the amines, the amphetamines have been shown to increase 5-HT release and turnover and it has been suggested that 5-HT is intimately involved in mediating many of the behavioral effects of the amphetamines [49].

## 3. Experimental Section

### 3.1. Animals and Treatment

Adult male Wistar rats (Harlan, S. Pietro al Natisone, UD, Italy) weighing 200–250 g were used. They were housed in standard cages in a controlled temperature room (22 ± 1 °C), and relative humidity (55 ± 5%) under a 12-h light/dark cycle (lights on from 8:00 AM to 8:00 PM). Standard laboratory chow and tap water were available *ad libitum*. Procedures involving animals and their care were conducted in conformity with the institutional guidelines of the Italian Ministry of Health (D.L. 116/92), the Declaration of Helsinki, the Guide for the Care and Use of Mammals in Neuroscience and Behavioral Research (National Research Council 2004), the Directive 2010/63/EU of the European Parliament and of the Council of 22 September 2010 on the protection of animals used for scientific purposes. All efforts were made to minimise the number of animals used and their suffering. Carvacrol (purity > 98%, Sigma Aldrich s.r.l., Milan, Italy) was dissolved in peanut oil and administered *per os*, by gavage, at different doses, (12.5 mg/kg for 7 consecutive days; 150 or 450 mg/kg for acute administration), accordingly to literature data and preliminary experience [18,22,23,31]. Control animals received oral administration of peanut oil (vehicle). Carvacrol and vehicle were administered in a volume of 1 mL/kg. Experimental procedures were performed 2h after acute doses; in the subchronic condition experiments were performed 2 h after last administration. Different sets of animals were used for neurochemical analysis (n = 8/group for acute and n = 8/group for subchronic experiments) and behavioural test (n = 10/group) for a total of 60 animals.

### 3.2. Measurement of Monoamine Neurotransmitter Levels

Rats were anesthetized intra-peritoneally with 3.6 mL/kg equithesin (composition: 1.2 g sodium pentobarbital; 5.3 g chloral hydrate; 2.7 g MgSO4; 49.5 mL propylene glycol; 12.5 mL ethanol and 58 mL distilled water), then were killed by decapitation and brains were immediately removed. For dissection, the brains were placed dorsal side up in an ice chilled rat brain matrix (World Precision Instruments, Inc. Sarasota, FL, USA) with slits spaced at 1 mm using an ice-chilled razor blade. Target regions were dissected out and weighted, according to the atlas of Paxinos and Watson [50]. Thereafter, PFC and HIPP were collected and immediately frozen on dry ice. Tissues were stored frozen at −80 °C until monoamine neurotransmitter levels quantification. At the time of analysis, samples were homogenized in 10 volumes (w/V) of perchloric acid 0.1 M. The homogenates were stored on ice for 30 min and then centrifuged at 10.000 x *g* for 10 min at 4 °C. The supernatants were then filtered and diluted before high performance liquid chromatography (HPLC) analysis.

DA and 5-HT concentrations were determined by HPLC coupled with an electrochemical detector (INTRO, Antec Leyden, The Netherlands). Amines separation was performed by a LC18 reversed phase cartridge column (Sphereclone, 150 mm × 2 mm, ODS 3 µm; Phenomenex, Castelmaggiore, Italy). The detection was accomplished by a Unijet cell (BASi, Kenilworth, U.K.) with a 6 mm diameter glassy-carbon electrode at a working potential of 0.65 V vs Ag/AgCl. The mobile phase used was 85 mM CH_3_COONa, 0.8 mM octanesulfonic acid, 0.3 mM EDTA, 15 mM NaCl, methanol 6%, in distilled water, buffered at pH 4.85. The flow rate was maintained by an isocratic pump (Shimadzu LC-10AD, Kyoto, Japan) at 0.220 mL/min. Data were acquired and integrated using Chromeleon software (version 6.60, Dionex, San Donato Milanese, Italy). Substrate concentration was expressed as fmol per milligram of wet tissue.

### 3.3. Forced Swimming Test

The test was performed according to Cryan *et al.*, [51]. Rats were individually forced to swim inside vertical Plexiglas cylinders (diameter 23 cm; height 70 cm) containing 30 cm of water maintained at 25 °C . During a preconditioning period, animals were placed into the cylinders and after 15 min in the water, they were removed and allowed to dry before being returned to their home cages. Twenty-four hours later, each rat was returned to the water-filled cylinder for 5 min and recorded by video camera. During the test sessions, the frequencies (5 min counts) of the following behaviours were measured: climbing (time spent in tentative of escaping), swimming (time spent moving around the cylinder) and immobility (time spent remaining afloat making only the necessary movements to keep its head above the water).

### 3.4. Statistical Analysis

Results are expressed as means + S.E.M. Statistical analyses were performed using Graph Pad 5.0 (GraphPad Software, San Diego, CA, USA) for Windows. Neurotransmitter concentrations were analysed using one-way analysis of variance (ANOVA) followed by Tukey’s multiple comparison test or unpaired student’s t-test. Behavioural data were analyzed by unpaired student’s t-test. Differences were considered statistically significant when P was less than 0.05.

## 4. Conclusions

In summary, our data demonstrated that carvacrol possess neuromodulatory properties in specific areas of the brain. Even though the specific mechanism through which carvacrol exerts its action and the functional meaning of the observed effects still remains unclear, our findings stimulate interest into deeply investigating this volatile compound. Since it is widely used not only as feed additive, but also as an essence for cosmetics and as a herbal remedy, further studies are required to establish its potential clinical efficacy as well as potential toxicity, before any recommendations can be made on its use.
